# Beyond castration: defining maximal testosterone control in advanced prostate cancer

**DOI:** 10.3389/fendo.2025.1634966

**Published:** 2025-10-01

**Authors:** Dongsheng Ma, Mengru Zhang, Xiaoguang Zhang, Tao Zhuo, Jianhong Xi

**Affiliations:** ^1^ Department of Reproductive Medicine, The Affiliated Bozhou Hospital of Anhui Medical University, Bozhou, China; ^2^ Department of Reproductive Medicine, The People’s Hospital Bozhou, Bozhou, China; ^3^ Reproductive male laboratory, The People’s Hospital Bozhou, Bozhou, China; ^4^ Department of Urology, The Fifth Affiliated Hospital of Xinjiang Medical University, Urumqi, China; ^5^ Department of Urology, The First Affiliated Hospital of Xinjiang Medical University, Urumqi, China

**Keywords:** testosterone, castration, advanced prostate cancer, androgen deprivation therapy, testosterone maximal control

## Abstract

**Objective:**

This study aimed to investigate the correlation between the minimum testosterone (T) level achieved during androgen deprivation therapy (ADT) for advanced prostate cancer and progression and prognosis. And to establish the new recommended threshold for defining castration-level testosterone.

**Methods:**

A retrospective analysis was conducted on 425 patients with advanced prostate cancer undergoing ADT. Patients were stratified into three groups based on their lowest testosterone level: castration low<10 ng/dL, castration 10–50 ng/dL, Non-castrated >50 ng/dL. To further explore subgroup progression and survival differences in low castrated testosterone levels, those castrated low testosterone levels were divided into two groups, castration ultra-low 5–10 ng/dL and castration extreme low<5ng/dL. Additionally, a small cohort (N = 29) of surgically castrated patients was included for subgroup analysis. Correlations between the minimum testosterone level and outcomes, time to progression (TTP) and overall survival (OS).

**Results:**

Significant differences in TTP were observed among the three groups (*P*<0.001), and both two groups (*P*<0.001). The castration low T level group had TTP of 24.62 ± 13.62 months and the lowest percentage of TTP<18 months (33.88%), the castration T level group had TTP of 15.65 ± 9.16 months with the second highest percentage of TTP<18 months (64.34%), the non-castrated T level group had TTP of 10.93 ± 7.89 months with the highest percentage of TTP<18 months (83.33%). There was a significant difference in survival rates between the three groups (*P*<0.001). Differences were found between the both two groups (*P*<0.01), with the castration low T level group demonstrating superior 3- and 5-year survival rates compared to the other groups. The non-castrated T level group had the worst prognosis. No significant differences in TTP or survival rates were observed between the castration ultra-low and extreme-low T subgroups. However, surgically castrated patients exhibited the poorest prognosis. Minimum testosterone level was weakly negatively correlated with TTP (r = -0.32, *P*< 0.001), but not significantly correlated with OS.

**Conclusion:**

Challenging the traditional castration standard, this study identifies 10 ng/dL (versus 50 ng/dL) as the critical testosterone threshold for evaluating tumor progression and prognosis in advanced prostate cancer patients on ADT.

## 1 Introduction

Prostate cancer (PCa) is a prevalent male genitourinary malignancy characterized by an insidious onset and indolent progression. The incidence of PCa exhibits substantial geographic heterogeneity, with higher rates observed in developed regions including North America, Australia, and Northern Europe, contrasting with lower rates in developing areas such as Southeast Asia (1). This disparity likely reflects contributions from genetic predisposition, family history, environmental factors, and lifestyle (2, 3). Globally, PCa incidence has risen overall while mortality has declined; notably, incidence displays greater spatial and temporal variation than mortality (4). The reduction in PCa mortality, however, is predominantly evident in economically advanced nations, with the most pronounced decreases occurring in high-income countries. These trends are attributable to the widespread adoption of prostate specific antigen (PSA) screening (impacting incidence) and therapeutic advancements (reducing mortality) (5–7). PCa incidence correlates strongly with age, exhibiting a progressive increase in older populations (8, 9). Prostate cancer demonstrates familial aggregation, genetic susceptibility, diverse histopathological subtypes, and heterogeneous treatment responses. Androgen deprivation therapy (ADT) constitutes the cornerstone of endocrine treatment for PCa and is utilized across cancer stages. Both gonadotropin-releasing hormone (GnRH) agonists and antagonists are established standard agents for ADT, achieving castration-equivalent testosterone suppression, pharmacological castration throughout ADT is generally comparable to surgical castration in efficacy (10). Testosterone plays a critical role in advanced PCa (aPC), traditionally, castration testosterone level is<50ng/dL, but beyond castration, redefining maximal testosterone control in aPC.

## 2 Methods

### 2.1 Study population

The study retrospectively analyzed the PCa follow-up databases of The Affiliated Bozhou Hospital of Anhui Medical University and The First Affiliated Hospital of Xinjiang Medical University (N = 1526, 2012–2023). Based on predefined diagnostic, inclusion, and exclusion criteria, 425 patients with advanced PCa were enrolled. Additionally, an external comparator cohort of surgically castrated patients (bilateral orchiectomy plus flutamide or bicalutamide; N = 29) was included for out-of-group analysis.

### 2.2 Study indicators

Clinical data: age, nationality, smoking history, alcoholism history, hypertension, diabetes.

Tumour characteristics: initial PSA, prostate volume, tumour stage, perineural invasion and visceral metastasis, Gleason score, tumour load.

Testosterone indicators: initial testosterone, testosterone at 1 month of ADT, minimum testosterone during ADT, testosterone response, testosterone escape, duration of ADT treatment, ADT dosage form.

Tumour progression indicators: time to progression (TTP), time to progression to metastatic castration resistant prostate cancer (mCRPC) or metastatic resistant prostate cancer (mRPC).

Survival indicators: overall survival (OS).

### 2.3 Study definitions

Advanced prostate cancer: regional and extra-regional distant metastases.

Advanced hormone sensitive prostate cancer (aHSPC): prostate cancer responsive to ADT.

Castration resistant prostate cancer (CRPC) (11): castration, serum testosterone (T)<50ng/dL; combined with one of the following conditions: PSA progression and imaging progression.

Tumour load: based on the CHAARTED study standard, it can be divided into high and low tumour load, with high tumour load defined as ≥4 bone metastases (including at least one vertebral or extra-pelvic metastasis) or visceral metastases (11).

Perineural invasion (PNI): pathological histological microscopy of prostate cancer shows that the tumour invades adjacent nerve tissue.

Visceral metastasis: biopsy of suspected metastatic tissue or conventional imaging CT or MRI confirmation.

In this study, advanced prostate cancer progression was defined as transition from aHSPC to mCRPC/mRPC.

### 2.4 Study inclusion and exclusion criteria

#### 2.4.1 Inclusion criteria

(1) Histologically confirmed prostate adenocarcinoma with clinical/radiological diagnosis of aPC (AJCC 8th edition staging): Regionally advanced (N_1_) and distant metastases (M_1_). (2) No prior androgen targeting therapy (ADT, combined androgen blockade, or novel hormonal therapy). (3) Serial testosterone monitoring.

#### 2.4.2 Exclusion criteria

(1) Concurrent other malignancy at diagnosis. (2) Severe comorbidities (cardiovascular, cerebrovascular, or psychiatric disorders). (3) Missing ≥ 2 categories of core data (clinical parameters, tumor characteristics, or testosterone metrics). (4) Loss to follow-up or withdrawal of consent.

### 2.5 Study methods

In the study, serum testosterone was quantified via electrochemiluminescence immunoassay (ECLIA). Patients were stratified by ADT-induced testosterone nadir. The lowest values of testosterone with ADT were grouped into three groups: castration low (<10 ng/dL), castration (10–50 ng/dL), and non-castrated testosterone level (>50 ng/dL). To further explore subgroup progression and survival differences in low castrated testosterone levels, those below 10ng/dL, were divided into two groups, castration ultra-low testosterone level 5-10ng/dL and castration extreme low testosterone level<5ng/dL. In addition, the study included data from an external cohort (N = 29) of patients who underwent surgical castration (bilateral orchiectomy) combined with flutamide or bicalutamide, with a default testosterone level of 0ng/dL and basically no testosterone level, approximates maximal androgen suppression, this approximation of the idealised physiological environment in which surgical castration is combined with medication, ignoring the potential impact of extratesticular testosterone microhormones on the study. Meanwhile, one-month testosterone value with ADT and minimum testosterone values of ADT were explored for correlation with TTP and OS.

### 2.6 Ethical review

It was a retrospective study, coded to anonymise identifiable information of the study subjects, and written informed consent for participation was not required from the participants or the participants’ legal guardians/next of kin. Ethical approval numbers: BYLS2024-147 (Medical Ethics Committee of The Affiliated Bozhou Hospital of Anhui Medical University), K202504-46 (Medical Ethics Committee of The First Affiliated Hospital of Xinjiang Medical University).

### 2.7 Statistical methods

SPSS 23.0 and R 4.4.2 statistical software were used for data processing and analysis. Measurement data were described by $\bar{x}\pm s$ , and t-test was used for comparison between groups, and count data were described by rate and χ^2^ test for comparison between groups. Survival time was calculated from the beginning of receiving treatment after diagnosis and was measured in months. Kaplan-Meier method was used to calculate the median survival of each group, and the survival rate of different time periods (1 year, 3 years, 5 years), and the survival curves were plotted, and the *P* value was calculated by comparing the differences between groups using Log-rank. Correlation analysis between two continuous measures was described by Pearson’s correlation with a confidence interval of 0.95. The test level for statistical analysis was α=0.05.

### 2.8 Study flow chart

**Figure 1:**
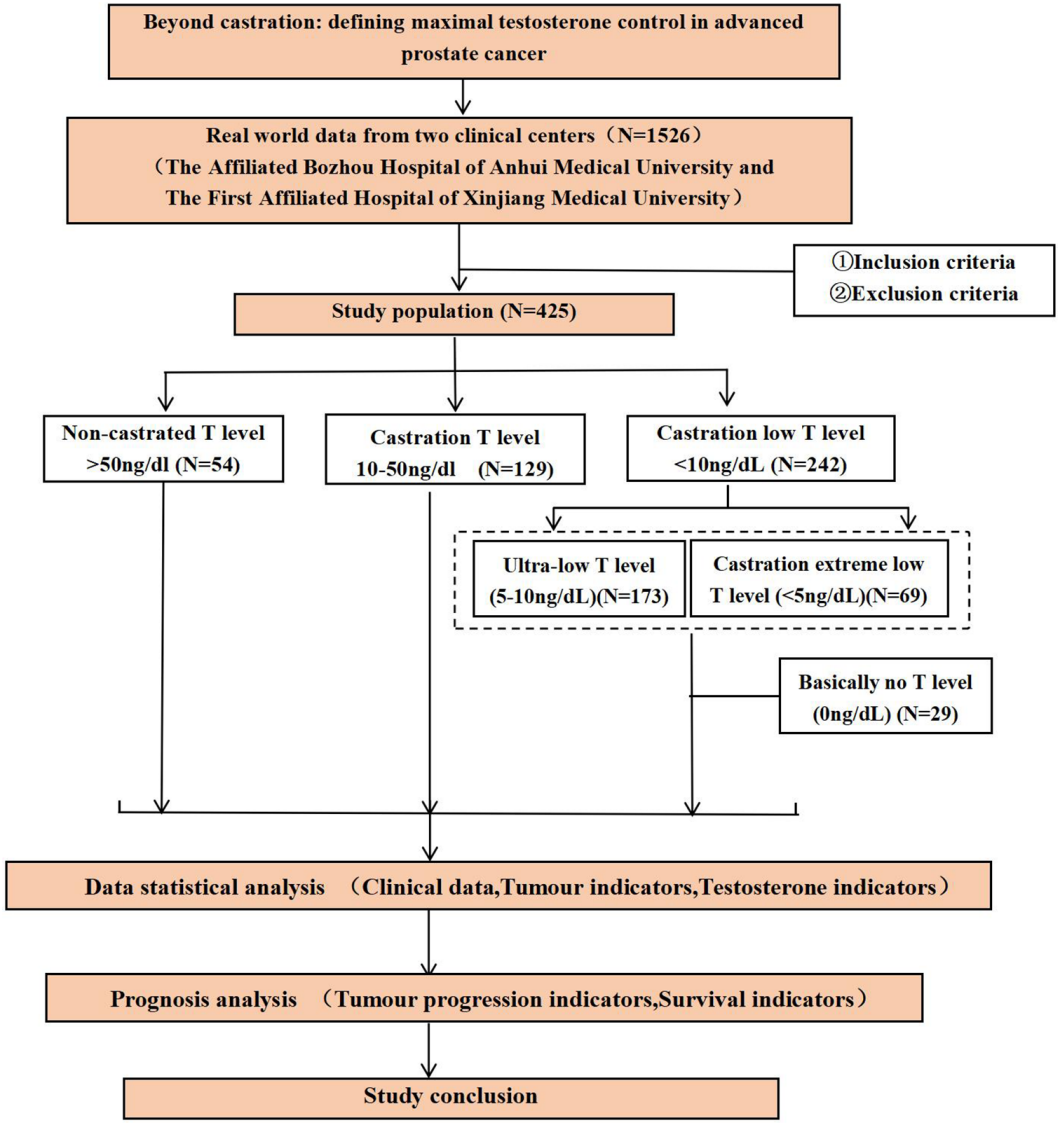
Study flow chart.

Participant screening, grouping, and analytical workflow are summarized in **Figure 1**.

## 3 Results

### 3.1 Intergroup comparison of characteristics among patients with advanced prostate cancer

The study population of 425 patients with advanced prostate cancer in the ADT treatment stage of testosterone minimum values was grouped into three groups: castration low testosterone level, castration testosterone level and non-castrated testosterone level. There was no significant difference in the age distribution of the groups (*P* = 0.629). There were differences in visceral metastasis and tumour load in the inter-group comparisons (*P* = 0.003, *P* = 0.002) and in the two by two comparisons between groups (*P*<0.05). Comparisons were significantly different. The non-castrated testosterone level was highest in visceral metastasis (27.78%) and tumour high load (74.07%). See **Table 1**.

**Table 1:** Baseline characteristics of advanced prostate cancer patients.

Characteristics	Groups				χ^2^	*P*
	Overall N = 425	Castration low T level N = 242	Castration T level N = 129	Non-castrated T level N = 54		
Age					0.93	0.629
<73	204 (48.00%)	116 (47.93%)	65 (50.39%)	23 (42.59%)		
≥73	221 (52.00%)	126 (52.07%)	64 (49.61%)	31 (57.41%)		
Nationality					0.68	0.713
Han	321 (75.53%)	180 (74.38%)	98 (75.97%)	43 (79.63%)		
Minority	104 (24.47%)	62 (25.62%)	31 (24.03%)	11 (20.37%)		
Smoking history					4.39	0.111
No	325 (76.47%)	179 (73.97%)	107 (82.95%)	39 (72.22%)		
Yes	100 (23.53%)	63 (26.03%)	22 (17.05%)	15 (27.78%)		
Alcoholism history					0.325	0.850
No	387 (91.06%)	219 (90.50%)	119 (92.25%)	49 (90.74%)		
Yes	38 (8.94%)	23 (9.50%)	10 (7.75%)	5 (9.26%)		
Diabetes					5.33	0.070
No	314 (73.88%)	182 (75.21%)	99 (76.74%)	33 (61.11%)		
Yes	111 (26.12%)	60 (24.79%)	30 (23.26%)	21 (38.89%)		
Hypertension					4.74	0.094
No	249 (58.59%)	137 (56.61%)	73 (56.59%)	39 (72.22%)		
Yes	176 (41.41%)	105 (43.39%)	56 (43.41%)	15 (27.78%)		
Gleason score					4.25	0.119
<9	210 (49.41%)	127 (52.48%)	54 (41.86%)	29 (53.70%)		
≥9	215 (50.59%)	115 (47.52%)	75 (58.14%)	25 (46.30%)		
Prostate volume (ml)					0.87	0.646
<55	212 (49.88%)	120 (49.59%)	62 (48.06%)	30 (55.56%)		
≥55	213 (50.12%)	122 (50.41%)	67 (51.94%)	24 (44.44%)		
Initial PSA (ng/ml)					4.44	0.108
<160	212 (49.88%)	128 (52.89%)	64 (49.61%)	20 (37.04%)		
≥160	213 (50.12%)	114 (47.11%)	65 (50.39%)	34 (62.96%)		
Perineural invasion					0.245	0.885
No	303 (71.29%)	172 (71.07%)	91 (70.54%)	40 (74.07%)		
Yes	122 (28.71%)	70 (28.93%)	38 (29.46%)	14 (25.93%)		
Visceral metastasis					11.97	0.003
No	356 (83.76%)	215 (88.84%)	102 (79.07%)	39 (72.22%)		
Yes	69 (16.24%)	27 (11.16%)	27 (20.93%)	15 (27.78%)		
Tumour stage					1.211	0.546
<3a	94(22.12%)	54 (22.31%)	31 (24.03%)	9 (16.67%)		
≥3a	331 (77.88%)	188 (77.69%)	98 (75.97%)	45 (83.33%)		
Tumour load					12.62	0.002
Low	168 (39.53%)	113 (46.69%)	41 (31.78%)	14 (25.93%)		
High	257 (60.47%)	129 (53.31%)	88 (68.22%)	40 (74.07%)		

Testosterone 1 month after ADT, ADT time, ADT continuity and ADT dosage form, were significantly different between groups (all *P*<0.01), and there were significant differences in two-by-two comparisons between groups (all *P*<0.001). See **Table 2**.

**Table 2:** Advanced prostate cancer patients ADT characteristics.

Characteristics	Groups				χ^2^	*P*
	Overall N = 425	Castration low T level N = 242	Castration T level N = 129	Non-castrated T level N = 54		
Testosterone 1 month after ADT(ng/dL)					134.67	<0.001
<20	211 (49.65%)	177 (73.14%)	34 (26.36%)	0 (0)		
≥20	214 (50.35%)	65 (26.86%)	95 (73.64%)	54 (100.00%)		
ADT time (months)					30.56	<0.001
<12	200 (47.06%)	93 (38.43%)	64 (49.61%)	43 (79.63%)		
≥12	225 (52.94%)	149 (61.57%)	65 (50.39%)	11 (20.37%)		
ADT continuity					28.79	<0.001
Intermittent	215 (50.59%)	104 (42.98%)	66 (51.16%)	45 (83.33%)		
Continuous	210 (49.41%)	138 (57.02%)	63 (48.84%)	9 (16.67%)		
ADT dosage form					12.37	0.002
Short dose (3.6mg)	271 (63.76%)	141 (58.26%)	85 (65.89%)	45 (83.33%)		
Long dose (10.8mg)	154 (36.24%)	101 (41.74%)	44 (34.11%)	9 (16.67%)		

Tumour progression between different groups, TTP classification and time were significantly different between three groups (*P*<0.001), and significant difference between both groups (*P*<0.001). Specifically, the castration low T level group had TTP of 24.62 ± 13.62 months and the lowest percentage of TTP<18 months (33.88%), the castration T level group had TTP of 15.65 ± 9.16 months with the second highest percentage of TTP<18 months (64.34%), the non-castrated T level group had TTP of 10.93 ± 7.89 months with the highest percentage of TTP<18 months (83.33%). The lowest minimum testosterone values increased during ADT with a decreasing trend in TTP. See **Tables 3**, **4**.

**Table 3:** Advanced prostate cancer patients tumour progression classified characteristic.

Characteristic	Groups				$\chi^{2}$	*P*
	Overall N = 425	Castration low T level N = 242	Castration T level N = 129	Non-castrated T level N = 54		
TTP (months)					59.70	<0.001
<18	210 (49.41%)	82 (33.88%)	83 (64.34%)	45 (83.33%)		
≥18	215 (50.59%)	160 (66.12%)	46 (35.66%)	9 (16.67%)		

**Table 4:** Advanced prostate cancer patients tumour progression time characteristics.

Characteristic	Group			t	*P*
	Castration low T level N = 242	Castration T level N = 129	Non-castrated T level N = 54		
TTP (months)	24.62 ± 13.62	15.65 ± 9.16	10.93 ± 7.89	103.07	<0.001

### 3.2 Intergroup comparison of survival among patients with advanced prostate cancer

**Figure 2:**
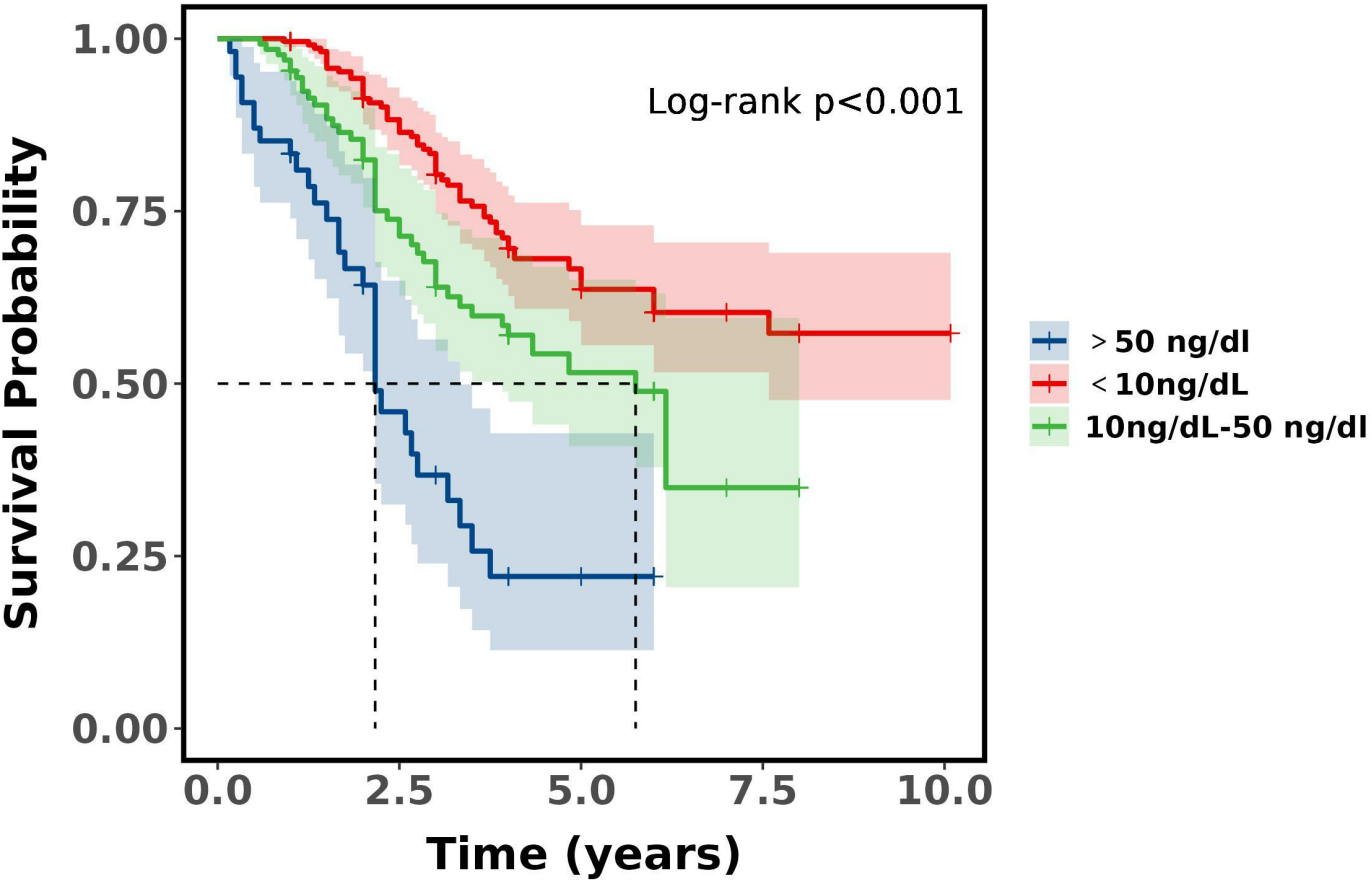
Survival curves between groups of patients with aPC.

There was a significant difference in survival rates between the three groups of castrated low, castrated and non-castrated T level (*P*<0.001), and a significant difference between both groups (*P*<0.001). Castration low T level had significantly higher survival rates than the other two groups at both 3 and 5 years, and non-castrated T level had the worst prognosis compared to the other two groups. Compared castration T level and non-castrated T level, the median 5-year survival rate was 63.67% for castration low T level, and the median 3-year survival rate was 80.30%, which proves that with ADT castration minimum testosterone less than 10ng/dL has a survival advantage over 10-50ng/dL. See **Table 5** and **Figure 2**.

**Table 5:** Kaplan-meier Estimates between groups of patients with aPC (95% CI).

Characteristics	1-year	3-year	5-year	*P*
Overall	96.24% (94.44%, 98.06%)	70.23% (65.29%, 75.53%)	55.18% (48.99%, 62.14%)	
Group				<0.001
Non-castrated T level (>50ng/dL)	83.33% (73.96%, 93.89%)	36.73% (23.92%, 56.41%)	22.04% (11.35%, 42.79%)	
Castration T level (10-50ng/dL)	95.35% (91.78%, 99.05%)	63.98% (54.75%, 74.76%)	51.60% (40.92%, 65.05%)	
Castration low T level (<10ng/dL)	99.59% (98.78%, 100.00%)	80.30% (74.66%, 86.36%)	63.67% (55.56%, 72.95%)	

### 3.3 Intersubgroup comparison of survival with advanced prostate cancer

Castration low T level (<10ng/dL) was divided into two groups, castration ultra-low testosterone level (5-10ng/dL) and castration extreme low testosterone level (<5ng/dL).And the study inclusion of surgical castration (bilateral orchiectomy) combined flutamide or bicalutamide in an external cohort (N = 29), the default testosterone 0ng/dL, basically no testosterone level,to compare whether there is a difference in progression and survival in prostate cancer.The tumour progression between the different subgroups did not show any significant difference in TTP time between the groups (*P* = 0.794), nor between the both two groups (*P*>0.05). See **Table 6**.

**Table 6:** TTP time characteristics in subgroup of patients with advanced prostate cancer.

	Groups				
Characteristic	Castration extreme low T level N = 173	Castration ultra-low T level N = 69	Basically no T level N = 29	t	*P*
TTP (months)	24.99 ± 13.00	23.70± 15.12	25.07± 14.89	0.23	0.794

**Figure 3:**
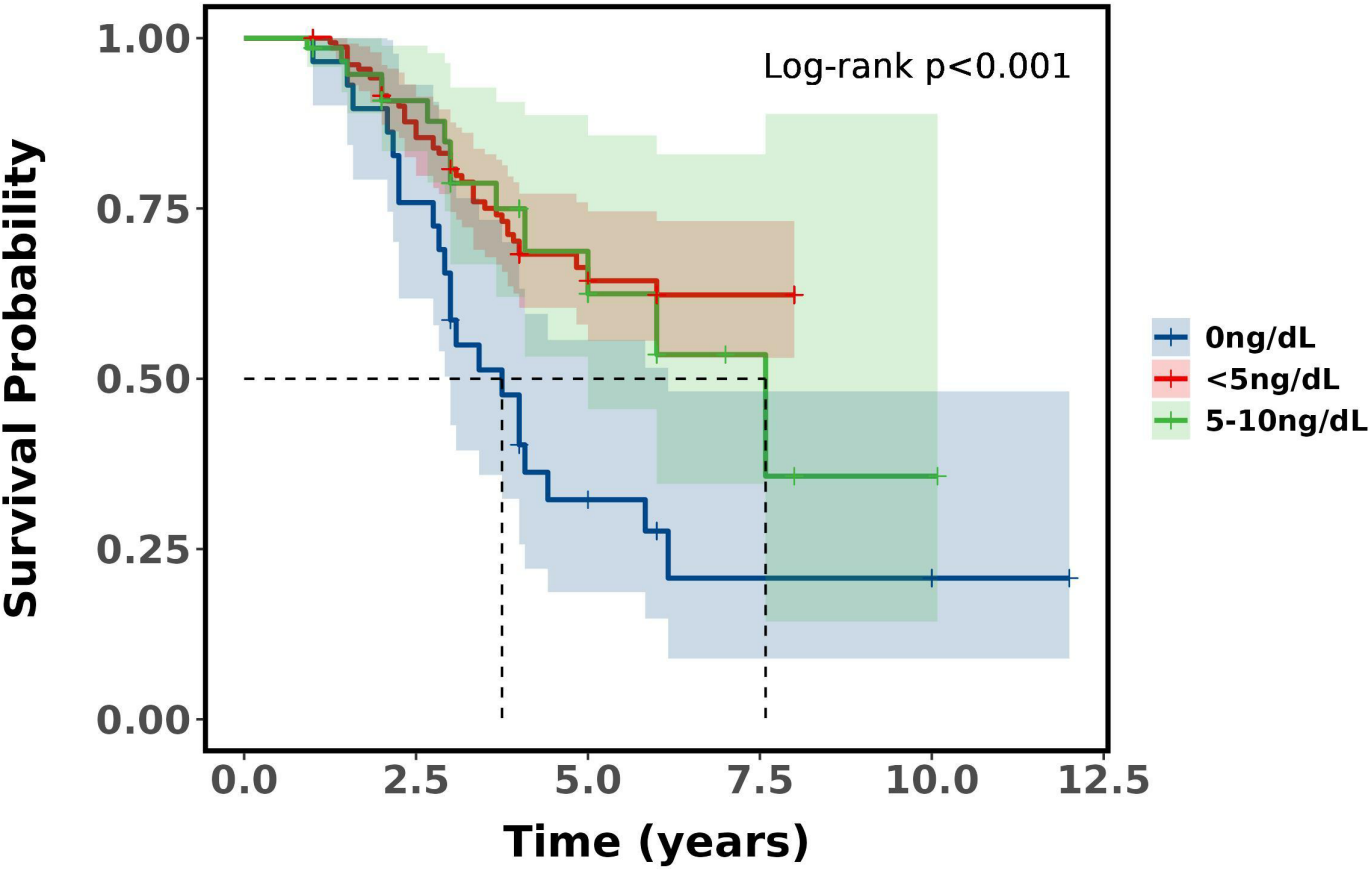
Survival curves between subgroups of patients with aPC.

There was a difference in survival between the Castration extreme low T level, castration ultra-low and basically no T level groups (*P*<0.001). Castration extreme low and castration ultra-low T level (*P* = 0.700), castration ultra-low and basically no T level (*P* = 0.022), castration extreme low and basically no T level (*P*<0.001). Castration extreme low and castration ultra-low T level did not differ in 3 and 5-year survival, whereas basically no T level had the worst prognosis compared to the other two groups, with a median 5-year survival was 32.24% and median 3-year survival was 58.62%, which demonstrates that with ADT castration with a minimum testosterone level of less than 5ng/dL has no survival difference over 5-10ng/dL, but surgical castration has the worst prognosis. See **Table 7** and **Figure 3**.

**Table 7:** Kaplan-Meier Estimates between subgroups of patients with advanced prostate cancer (95% CI).

Characteristics	1-year	3-year	5-year	*P*
Overall	99.26% (98.25%, 100.00%)	77.10% (71.56%, 83.08%)	58.15% (50.59%, 66.85%)	
Group				<0.001
Castration extreme low T level (<5ng/dL)	100.00% (100.00%, 100.00%)	80.79% (74.48%, 87.63%)	64.38% (55.58%, 74.58%)	
Castration ultra-low T level (5-10ng/dL)	98.55% (95.77%, 100.00%)	78.71% (66.80%, 92.75%)	62.47% (45.53%, 85.72%)	
Basically no T level (0ng/dL)	96.55% (90.13%, 100.00%)	58.62% (43.18%, 79.59%)	32.24% (18.67%, 55.69%)	

### 3.4 Analysis of the correlation between advanced prostate cancer progression, prognosis, and testosterone at different stages of ADT

**Figure 4:**
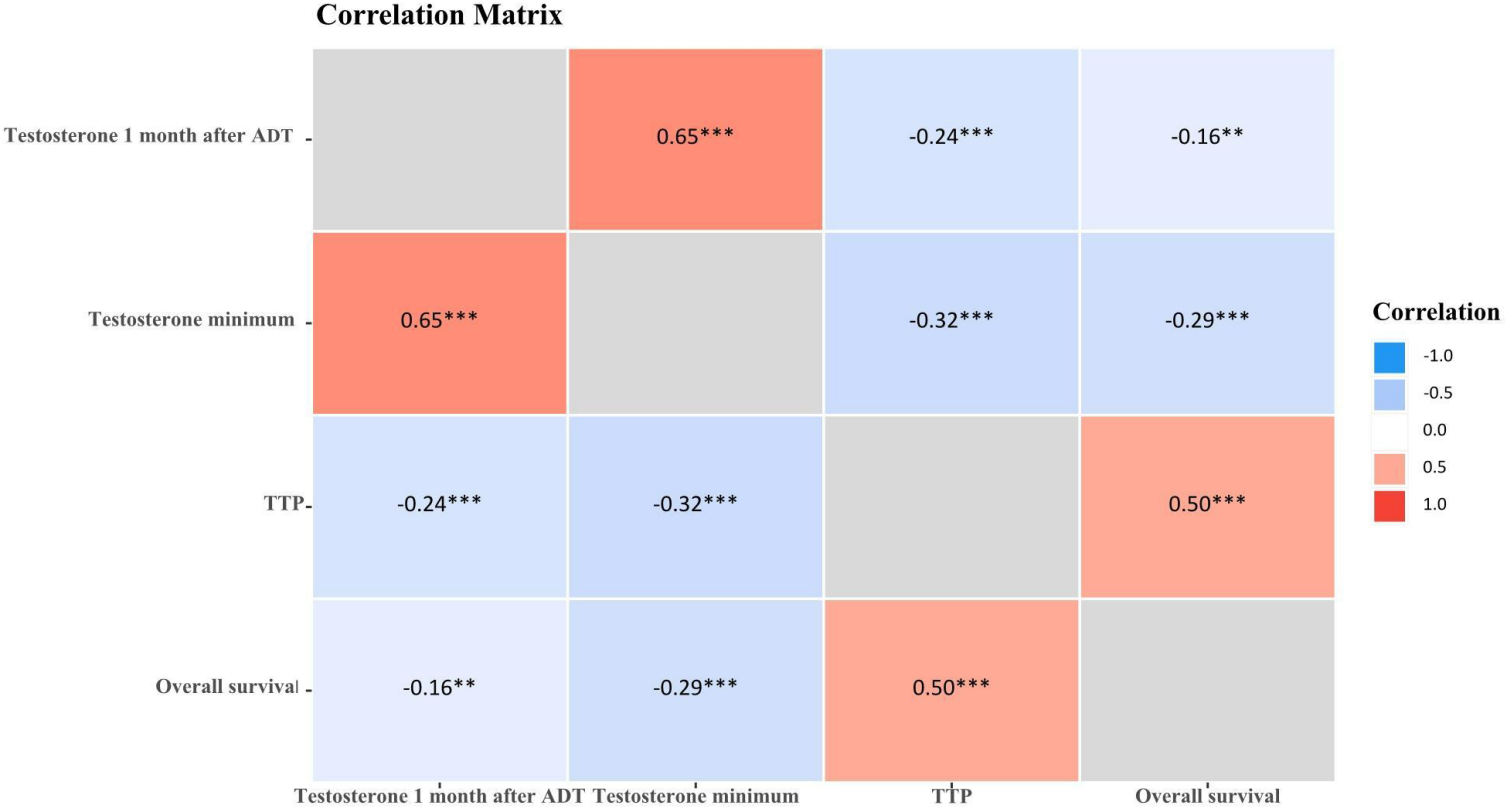
Correlation matrix of testosterone and prostate cancer advancement. *P<0.05; **P<0.01; ***P<0.001.

The correlation analysis of Testosterone minimum and time to progression showed that there was a weak negative correlation (r=-0.32, *P*<0.001), no negative correlation between testosterone minimum and OS (r=-0.29, *P*<0.001). While there was a medium positive correlation between testosterone 1 month after ADT and testosterone minimum (r=0.65, *P*<0.001), there was a medium positive correlation between TTP and overall survival (r=0.50, *P*<0.001), See **Table 8** and **Figure 4**.

**Table 8:** Correlation analysis results of testosterone and prostate cancer advancement.

Parameter A	Parameter B	r	95%CI	t	*P*
Testosterone 1 month after ADT	Testosterone minimum	0.65	(0.58,0.71)	15.087	<0.001
Testosterone 1 month after ADT	TTP	-0.24	(-0.35,-0.14)	-4.433	<0.001
Testosterone 1 month after ADT	Overall survival	-0.16	(-0.27,-0.05)	-2.898	0.004
Testosterone minimum	TTP	-0.32	(-0.41,-0.21)	-5.877	<0.001
Testosterone minimum	Overall survival	-0.29	(-0.39,-0.18)	-5.317	<0.001
TTP	Overall survival	0.50	(0.41,0.58)	10.082	<0.001

## 4 Discussion

Prostate cancer exhibits high androgen dependence, with testosterone driving tumor growth; thus, ADT constitutes the therapeutic cornerstone. No significant intergroup differences existed in age distribution (*P* = 0.629) or Gleason scores (*P* = 0.119), castration low, castration and non-castrated, suggesting minimal influence of these factors on testosterone response to ADT. Approximately half of patients were aged >73 years—notably, PCa mortality risk increases from 17% (diagnosis age<70) to 21% (≥70) (12). Age independently predicts inferior survival post-ADT, with significantly better outcomes in patients aged 71–75 versus >75 years (13). Visceral metastasis and tumour load differed significantly between groups (both *P*<0.05), predominating in the non-castrate cohort (27.78% and 74.07%, respectively). This also implies that visceral metastasis and tumour load seem to have a relatively important effect on the sensitivity of testosterone response with ADT treatment, especially when extra-regional visceral metastasis and high tumour load have occurred. ADT parameters (Testosterone 1 month after ADT, ADT time, ADT continuity and ADT dosage form) also varied substantially (all *P*<0.01). Intermittent ADT (IADT) prolongs median time to mCRPC versus continuous ADT (CADT) (14). In aPC, IADT can produce oncologic outcomes similar to CADT. In terms of overall survival, the hazard ratios for IADT and CADT were very similar (range: 0.98-1.08) (15). Maintaining serum testosterone<20–30 ng/dL extends ADT response (16).

Tumour progression between different groups, TTP classification and time were significantly different between groups (*P*<0.001), and significant difference between both groups (*P*<0.001). Specifically, the castration low T level group had TTP of 24.62 ± 13.62 months and the lowest percentage of TTP<18 months (33.88%), the castration T level group had TTP of 15.65 ± 9.16 months with the second highest percentage of TTP<18 months (64.34%), the non-castrated T level group had TTP of 10.93 ± 7.89 months with the highest percentage of TTP<18 months (83.33%). Lower nadir testosterone correlated with longer TTP. The 18-month threshold reflects typical transition to castration resistance, because PCa is mostly hormone-sensitive at first diagnosis, but tends to progress to castration resistance after 18–36 months of endocrine therapy. ADT is the standard of treatment for patients with aPC. However, the castration serum testosterone threshold, as well as the time to progression to mCRPC in patients with newly diagnosed aPC remain controversial. While<50 ng/dL does not predict ADT efficacy, testosterone ≤25 ng/dL at 1 month optimally predicts prolonged TTP (adjusted HR: 1.46; 95% CI: 1.08–1.96; *P* = 0.013) *(* 17). Paradoxically, lower baseline testosterone (<12 nmol/L) associates with earlier mCRPC development (19.0 vs. 22.4 months; *P* = 0.031) *(* 18). There was a significant difference in survival rates between three groups (*P*<0.001), and significant difference between both groups (*P*<0.001). Castration low T level had significantly higher survival rates at both 3 years (median survival rate,80.30%) and 5 years (median survival rate,63.67%), and non-castrated T level had the worst prognosis. However the exact relationship between testosterone levels and the prognosis of PCa remains under-explored. Prognostically, low diagnostic testosterone (2.0–8.0 nmol/L) independently predicts reduced OS (19). Low serum testosterone level (<450ng/dL) in metastatic hormone sensitive prostate cancer (mHSPC) patients with ADT predicted a poor prognosis, and CRPC-free survival and overall survival in the low-testosterone group were significantly shorter than those in the high-testosterone group (*P* = 0.021 and *P*<0.001) *(* 20). Lower baseline serum testosterone (<250ng/dL) was significantly associated with poorer survival outcomes in patients with first-treatment mHSPC undergoing CADT (21). Minimum testosterone of 20 ng/dL is the most significant critical level for overall survival in PCa with ADT therapy (22, 23). However it has also been suggested that a minimum testosterone value of 30 ng/dL provides the best overall sensitivity and specificity for predicting death. Serum testosterone level<30 ng/dL was associated with a significantly lower risk of death (adjusted HR,0.45;95%CI,0.22-0.94; *P* = 0.034) *(* 24). In this study, it was concluded that in patients with aPC, with ADT testosterone minimum set at 10 ng/dL seems to be more appropriate and the clinical survival benefit is more favourable. In addition, the time to arrival of testosterone nadir tends to be about 1 year or less. The rate of descent to minimum testosterone early in ADT treatment (6 months) was categorised as rapid or slow, with no significant difference in overall survival (22). Thus the key factor in prognosis is not the testosterone rapid decline, but whether the lowest testosterone reaches below the desirable threshold.

Castration low T level (<10ng/dL) was divided into two groups, castration ultra-low (5-10ng/dL) and castration extreme low testosterone level (<5ng/dL). To assess whether maximal testosterone suppression (almost no testosterone) confers incremental benefit, the study inclusion of surgical castration (bilateral orchiectomy) combined with flutamide or bicalutamide in a small sample of data, the default testosterone 0ng/dL, basically no testosterone level, to compare whether there is a difference in progression and survival in PCa. The tumour progression between the different subgroups did not show any significant difference in TTP time between the groups (*P* = 0.794), nor between the two groups (*P*>0.05). With improved prognosis in patients with testosterone suppression below 20 ng/dL and even 10 ng/dL, but with ADT minimum testosterone<10ng/dL continued subgroup stratification appeared to make no difference for PCa progression. Testosterone breakthrough, or an elevation of testosterone above the castration threshold from the first month to the sixth month of ADT, is usually associated with inadequate ADT treatment or insensitive ADT treatment. The weighted mean testosterone breakthrough rate was significantly higher for the 20 ng/dL threshold compared with 50 ng/dL (41.3% vs 6.9%, *P*<0.0001), and clinical factors such as frequency of testosterone monitoring, testosterone test method and route of ADT administration did not significantly affect testosterone breakthrough rate (25). Early standard ADT reduces symptoms of cancer progression in advanced hormone-sensitive prostate cancer and may prolong progression-free survival and overall survival (26, 27).

The results of this study show a difference in survival between the castration extreme low T level (<5ng/dL), castration ultra-low T level (5-10ng/dL) and basically no T level (0ng/dL) groups (*P*<0.001). Castration extreme low T level and castration ultra-low T level did not differ in 3 and 5-year survival, whereas basically no T level had the worst prognosis compared to the other two groups, with a median 5-year survival was 32.24% and median 3-year survival was 58.62%, which demonstrates that with ADT castration with a minimum testosterone level of less than 5ng/dL has no survival difference over 5-10ng/dL, but surgical castration (complete androgen blockade, testosterone almost 0ng/dL) has the worst prognosis, suggesting that near-complete androgen suppression (0 ng/dL) may not improve outcomes, instead, it leads to a worse prognosis. The low testosterone response often depends not only on the individual sensitivity to androgen deprivation therapy, but also on the synergistic effects of combination therapy. Combination therapy based on ADT possesses efficacy and safety in mHSPC, and combination therapy achieves better survival outcomes than ADT alone (28). Early intensive treatment of mHSPC, especially in patients with high tumour load, is clinically recommended to be suitable for ADT-based chemotherapy triple therapy, which is more beneficial than double therapy for overall survival (29–31). The correlation analysis of testosterone minimum and time to progression showed that there was a weak negative correlation (r=-0.32, *P*<0.001), no negative correlation between testosterone minimum and OS (r=-0.29, *P*<0.001). While there was a medium positive correlation between testosterone 1 month after ADT and testosterone minimum (r=0.65, *P*<0.001), there was a medium positive correlation between TTP and OS (r=0.50, *P*<0.001). Further demonstrating that low testosterone represents maximal testosterone control, thereby slowing cancer progression. But in the complex environment of the real world, patients with aPC, priority must be given to determining which treatment combinations and sequences of treatment will be of greatest benefit (32). And taking into socio-personal, economic-temporal multiple factors and respecting self-selection.

It has been found that after castration treatment, 5-10% of pre-treatment androgen levels are often still present in prostate tissue, due to the fact that in addition to androgens produced by the testes, residual androgens are secreted by the adrenal glands and other glands. For blocking androgens, treatment with anti-androgen drugs is also required. Competitive inhibition of androgens, which cannot bind to tumour receptors, is known as maximal androgen blockade (MAB). Surgical castration is one of the ancient and traditional methods of MAB, and postoperative androgen levels can be rapidly reduced to the desired goal (ranges from 3 to 12 hours, average 8.6 hours) (33), serum testosterone level was approximately 15 ng/dL (34). The study also additionally included surgical castration (bilateral orchiectomy) combined with flutamide or bicalutamide. Surgical castration was defaulted to 0 ng/dL of testosterone in the study, and testosterone was only considered to be a single source from testes, not including exogenous testosterone replacement and extra-testicular. Testosterone replacement therapy is not routinely administered to all older men with low testosterone levels, but it is recommended that individualised treatment may be considered, when appropriate. For old men with low-risk PCa who have persistently low testosterone level and significant symptoms, risk-benefit analysis (35, 36).

**Figure 5:**
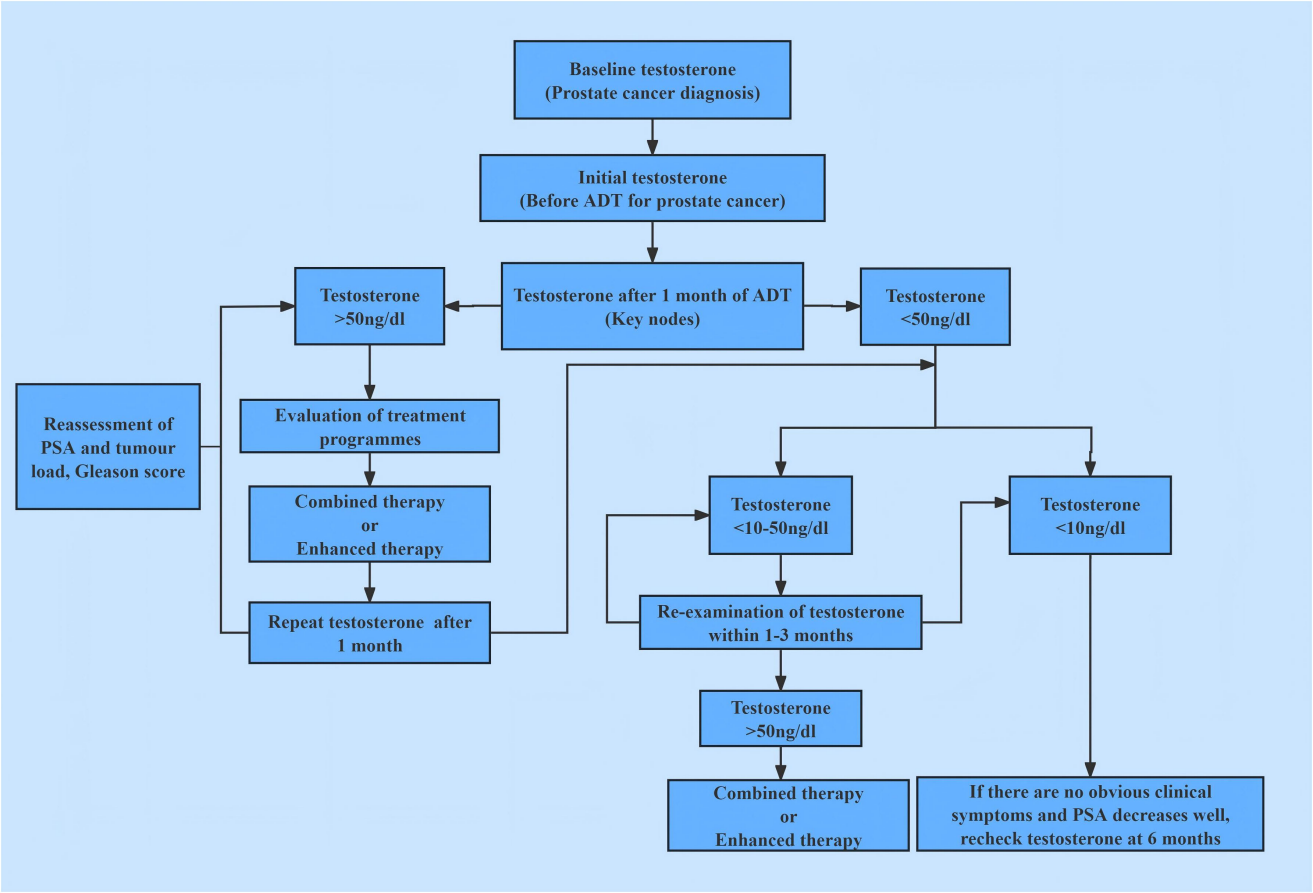
Schematic diagram of testosterone management process during ADT treatment.

In androgen deprivation therapy, the nadir serum testosterone level and its fluctuation range constitute independent prognostic factors distinct from traditional PSA metrics. Achieving and sustaining deeper, more stable testosterone suppression (recommended testosterone castration level 10 ng/dL) delays cancer progression and extends survival. This contrasts with the conventional view, where androgen deprivation therapy merely aims to reduce testosterone to castrated level (consensus testosterone castration level< 50 ng/dL). Instead, the present theory emphasizes ‘deep castration suppression’ (maximal control) and ‘long-term stability’ (dynamic stability), which also considers preserving the necessary, appropriate, sustained physiological requirement of testosterone. This study established the aPC treatment concept of changing from ‘testosterone castration’ to ‘testosterone maximal control’, promoting the development of standardized and individualized treatment mode for aPC patients. The study established a schematic diagram of testosterone management process, see **Figure 5**.

A nadir testosterone level of 10 ng/dL serves as a significant predictor of tumor progression and survival in patients with advanced PCa undergoing ADT. Challenging the conventional castration threshold of 50 ng/dL, our findings establish 10 ng/dL as a more discriminative and clinically relevant boundary for evaluating PCa outcomes. These results advocate for a refined definition of maximal testosterone control, moving beyond traditional castration benchmarks, to better reflect the level of testosterone control required to optimize prognosis in advanced PCa.

## Data Availability

The raw data supporting the conclusions of this article will be made available by the authors, without undue reservation.
